# Classification of Grades of Subchondral Sclerosis from Knee Radiographic Images Using Artificial Intelligence

**DOI:** 10.3390/s25082535

**Published:** 2025-04-17

**Authors:** Soo-Been Kim, Young Jae Kim, Joon-Yong Jung, Kwang Gi Kim

**Affiliations:** 1 Medical Devices R&D Center, Gil Medical Center, Gachon University, Incheon 21565, Republic of Korea; rlatnqls22ee@gmail.com; 2 Gachon Biomedical & Convergence Institute, Gil Medical Center, Gachon University, Incheon 21565, Republic of Korea; kimyj10528@gmail.com; 3 Department of Radiology, College of Medicine, The Catholic University of Korea, Seoul St. Mary’s Hospital, Seoul 06591, Republic of Korea; jjdragon112@gmail.com; 4 Department of Biomedical Engineering, College of Medicine, Gil Medical Center, Gachon University, Incheon 21565, Republic of Korea

**Keywords:** artificial intelligence, classification, convolutional neural network, deep learning, knee radiographs, subchondral sclerosis

## Abstract

Osteoarthritis (OA) is the most common joint disease, affecting over 300 million people worldwide. Subchondral sclerosis is a key indicator of OA. Currently, the diagnosis of subchondral sclerosis is primarily based on radiographic images; however, reliability issues exist owing to subjective evaluations and inter-observer variability. This study proposes a novel diagnostic method that utilizes artificial intelligence (AI) to automatically classify the severity of subchondral sclerosis. A total of 4019 radiographic images of the knee were used to train the 3-Layer CNN, DenseNet121, MobileNetV2, and EfficientNetB0 models. The best-performing model was determined based on sensitivity, specificity, accuracy, and area under the curve (AUC). The proposed model exhibited outstanding performance, achieving 84.27 ± 1.03% sensitivity, 92.46 ± 0.49% specificity, 84.70 ± 0.98% accuracy, and 95.17 ± 0.41% AUC. The analysis of variance confirmed significant performance differences across models, age groups, and sexes (*p* < 0.05). These findings demonstrate the utility of AI in diagnosing and treating knee subchondral sclerosis and suggest that this approach could provide a new diagnostic method in clinical medicine. By precisely classifying the grades of subchondral sclerosis, this method contributes to improved overall diagnostic accuracy and offers valuable insights for clinical decision-making.

## 1. Introduction

Osteoarthritis (OA) is the most prevalent joint disease, affecting over 300 million people worldwide or approximately 4% of the global population [1,2]. The global prevalence of OA is estimated to be 16% in individuals aged 15 years and older, and around 30% in those aged 60 years and above [3]. Notably, women are more susceptible to OA, and the incidence of OA is expected to increase with the increase in the aging population [4,5,6,7].

The image classification capabilities of deep learning have improved substantially with the development of convolutional neural networks (CNNs) [8]. The visual assessment of radiographic images for subchondral sclerosis is often subject to reliability issues due to subjective evaluations and interobserver variability [9,10]. To mitigate these uncertainties in diagnosis, AI-based research has been increasingly explored in the medical field [11] with promising outcomes observed in studies on the importance of knee OA feature analysis [12] and knee OA grading [13,14].

Tiulpin et al. proposed a deep learning-based approach for the automatic diagnosis of knee OA using plain radiographs, achieving a classification accuracy of 66.71% on the osteoarthritis initiative (OAI) and multicenter osteoarthritis study (MOST) datasets using the ResNet34 model [15]. Patron et al. utilized a ResNet50-32×4d model to study the tibial region, an early sign of arthritis, and achieved an overall accuracy of 86.9%, sensitivity of 90.9%, and specificity of 75% using the OAI and MOST datasets [16]. Von Schacky et al. employed the RetinaNet model for hip OA feature grading using DenseNet to learn specific features, such as femoral osteophytes, acetabular osteophytes, joint space narrowing, subchondral sclerosis, and subchondral cysts, achieving subchondral sclerosis accuracy of 95.8% on an internal test set and 88.5% on an external test set [17]. Abdullah and Rajasekaran used Faster R-CNN to detect knee joint space width and AlexNet for classification, achieving an accuracy of 98.9% [18]. Yoon et al. leveraged OAI data for automatic knee position detection and tibial width measurement and developed an AI model (MediAI-OA) for Kellgren–Lawrence (KL) grade classification and osteophyte detection using the NASNet model, with an overall accuracy of 92% [19]. Gornale et al. developed an algorithm to calculate the cartilage area and thickness from knee radiographic images, achieving an accuracy of 99.81% with a K-NN classifier and 95.09% with a decision tree classifier [20]. Subha et al. proposed a dual convolutional neural network based on the Gaussian Aquila Optimizer, which achieved an accuracy of 98.77%, sensitivity of 98.25%, and specificity of 98.93% on 2283 knee images [21]. Mohammed et al. performed binary and three-class classifications of OA presence and severity using pre-trained models, with ResNet101 achieving maximum classification accuracies of 69%, 83%, and 89% for each task [22]. Mahum et al. conducted automatic OA detection and classification using the DenseNet pre-trained model on the OAI dataset, recording an accuracy of 98.22% [23]. Song et al. proposed KOA-CAD, integrating a Laplacian-based strategy and AMD-CNN for the detection and grading of knee OA using vibroarthrography and physiological signals, achieving an automatic detection accuracy of 93.6%, early detection accuracy of 92.1%, and grading detection accuracy of 84.2% [24]. Boniatis et al. introduced a classification ensemble for assessing the severity of hip OA by manually segmenting 64 regions of interests (ROIs) corresponding to the hip space and generating new texture features, achieving accuracies of 100% and 95.7% for normal/OA (stage 1) and mild/moderate-to-severe (stage 2) classifications, respectively [25]. Michael Fei et al. proposed a regression-based deep learning model using the EfficientNet architecture to predict continuous KL scores from radiographs, achieving an AUC of 0.83 [26].

Currently, the most widely used method for radiographic evaluation of osteoarthritis (OA) is the Kellgren–Lawrence (KL) grading system, which classifies OA severity on a scale from 0 (normal) to 4 (severe) based on radiographic images. However, the KL grade primarily focuses on osteophytes, which has been pointed out as a limitation [27]. For a more accurate assessment of OA, detailed investigations of specific features such as osteophytes, joint space narrowing, subchondral sclerosis, bone cysts, and joint deformity are needed. Although studies have attempted to quantify OA severity, research specifically analyzing subchondral sclerosis remains limited, and most existing studies only evaluate its presence or absence. Cooper et al. emphasized the importance of the reproducibility of individual component features in the development of radiographic systems for grading OA and reported low reliability in the assessment of subchondral sclerosis, leading to poor reproducibility [28]. Yoon et al. highlighted the need for the development of related models to evaluate OA features because the MediAI-OA model cannot assess subchondral sclerosis or bone abnormalities [19].

To address these issues, this study proposes a deep-learning-based radiograph data-driven classification model for knee subchondral sclerosis. The clinical utility of this model was validated by comparing it with the existing KL grading system using metrics such as sensitivity, specificity, accuracy, and AUC. Thus, we aimed to develop a more objective and precise evaluation method for subchondral sclerosis, thereby enhancing the reliability of the diagnosis and improving clinical decision support systems.

## 2. Methods

### 2.1. Data Collection and Labeling

In this study, 4019 knee joint radiographic images were collected from 4019 patients at the Catholic University Hospital. The dataset consisted of 643 men and 3376 women participants, with a mean age of 66.81 years (±11.83 years), ranging from 6 to 96 years. All the procedures conducted in this study adhered to the ethical principles outlined in the Declaration of Helsinki and were approved by the Institutional Review Board (IRB No. KC23RIDI0485).

**Figure 1:**
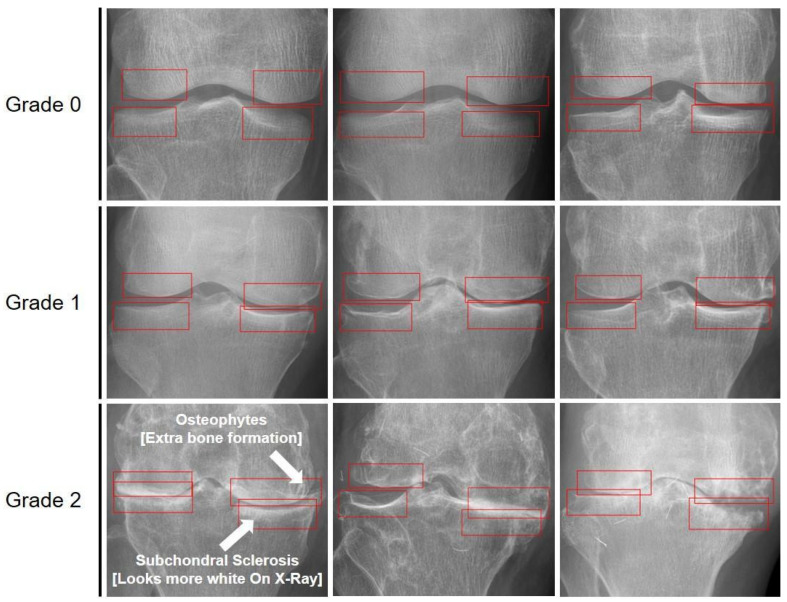
Subchondral labeling and images for each sclerosis grade. The red squares in the image highlight the ROI where subchondral sclerosis is most prominent. Grade 0: no abnormalities in the subchondral bone. Grade 1: mild sclerosis or partial destruction may be observed on the surface of the subchondral bone. Grade 2: significant sclerosis is present, with substantial destruction in multiple areas, potentially impacting joint function. The arrows in the grade 2 image indicate the areas of subchondral sclerosis and OA. These points represent the key lesions characteristic of grade 2 in this study, typically observed in more advanced stages of OA. Notably, subchondral sclerosis appears as white areas in X-ray images.

The ROI was delineated by a specialist on the knee radiographic images following the criteria illustrated in Figure 1. The labeling process was independently conducted and verified by two specialists. In cases in which different grades were assigned, the final decision was reached through consensus. Figure 1 presents examples of subchondral labeling and subchondral sclerosis grades, with the subchondral sclerosis areas highlighted. Grade 0 indicated no abnormalities in the subchondral region, Grade 1 indicated mild sclerosis or partial destruction of the subchondral bone surface, and Grade 2 indicated significant destruction that could impair joint function.

The dataset was distributed across three grades to avoid any imbalance: 1470 images in Grade 0, 1282 images in Grade 1, and 1267 images in Grade 2. Thereafter, the dataset was divided into training (80%), validation (10%), and testing (10%) datasets. This resulted in 3215 images for training and 402 images each for validation and testing. Table 1 provides a detailed breakdown of the data split according to subchondral sclerosis grades for the training, testing, and validation subsets. The distribution of subchondral sclerosis grades according to sex and age within the test dataset is presented in Table 2.

### 2.2. Training Environment and Data Preprocessing

The system used for training consisted of an NVIDIA GeForce GTX 1660 (NVIDIA, Santa Clara, CA, USA) graphics processing unit, an Intel Core i7 10700 CPU (Intel Corporation, Santa Clara, CA, USA), and 32 GB of RAM. Deep learning training was conducted on a Windows 10 Pro operating system using Python (Ver. 3.12.1) and the Keras framework (Ver. 2.10.0, with TensorFlow backend).

**Figure 2:**
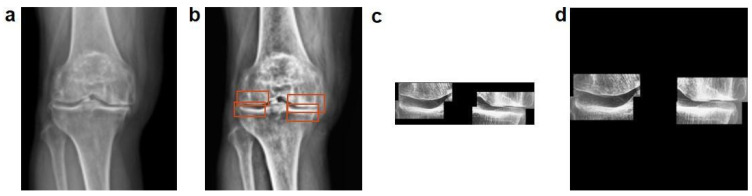
Data preprocessing steps. (**a**) Convert to 8-bit scale: this step involves adjusting the pixel values of the data to an 8-bit scale, reducing memory usage and facilitating easier data processing and analysis. (**b**) CROP: the ROI for subchondral sclerosis data is masked and cropped to the size of the relevant area for further use. (**c**) CLAHE (contrast limited adaptive histogram equalization): enhances image contrast to make the features of joint tissues more prominent. (**d**) Zero padding: preserves the spatial dimensions of the images to prevent distortion and improve training efficiency.

The DICOM images were converted to an 8-bit scale by adjusting the pixel value range to 0–255. This conversion was performed to reduce the memory usage and facilitate easier data processing and analysis. To study the subchondral regions, the ROI was designated as a mask to extract the relevant area from the images. Contrast limited adaptive histogram equalization (CLAHE), a contrast enhancement technique used in image processing, was applied to equalize the histogram of the image, thereby improving the local contrast of the images [29,30,31,32]. CLAHE was specifically employed to enhance the visibility of the features of subchondral sclerosis in the X-ray images. Finally, zero padding was applied to preserve the spatial dimensions of the images, prevent distortion, and improve training efficiency. Figure 2 illustrates the data preprocessing steps.

### 2.3. Subchondral Sclerosis Classification Models and Statistical Analysis

**Figure 3:**
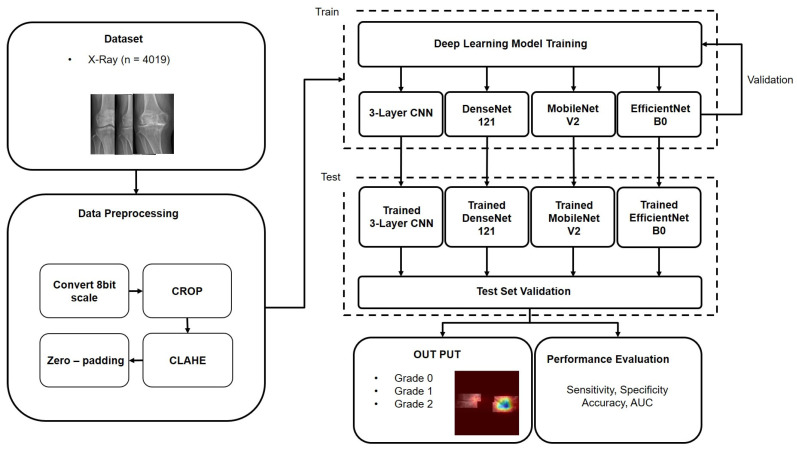
Workflow for subchondral sclerosis grading classification model training. Data collection, data preprocessing, model training and validation, and model evaluation.

Four deep learning models were employed for the classification of subchondral sclerosis grades: 3-Layer CNN, DenseNet, MobileNet, and EfficientNet. These models were selected based on their proven effectiveness in medical image analysis [33,34,35]. The 3-Layer CNN model utilizes a series of convolutional and pooling layers to sequentially extract features and perform classification. DenseNet features a densely connected network architecture in which each layer receives the output of all preceding layers as input, enhancing feature propagation and efficiency. MobileNet combines depth-wise separable convolutions and pointwise convolutions to significantly reduce the model size and computation and achieve model lightness and speed improvements. EfficientNet is designed to improve network performance and efficiency by balancing the model depth, width, and resolution. To prevent overfitting during training, dropout was applied at a fixed rate of 0.4. A seed size of 42 was set to ensure reproducibility of the training process. Each model was pretrained on the ImageNet dataset and then fine-tuned for the subchondral sclerosis grading classification. Common training parameters included a batch size of 32, 50 epochs, and an Adam optimizer with a learning rate of 0.00001. Figure 3 illustrates the overall workflow for training of subchondral sclerosis grading classification models used in this study.

To assess differences in subchondral sclerosis grades by age group and sex, analysis of variance (ANOVA) was performed, followed by Tukey’s HSD test to identify significant differences. Additionally, the Mann–Whitney U test was used to evaluate differences in subchondral sclerosis detection accuracy between specific groups. All statistical tests were conducted with a significance level set at *p*-value < 0.05.

## 3. Results

In this study, four models were trained to classify subchondral sclerosis grades using a radiographic dataset, and their performances were evaluated using a test set of 402 images. The best-performing model was selected based on a 10-fold cross-validation. The sensitivity, specificity, accuracy, and AUC values were calculated and are presented in Table 3. The AUC for the 3-Layer CNN, DenseNet121, MobileNetV2, and EfficientNetB0 models were 89.52 ± 0.46%, 94.68 ± 0.84%, 94.45 ± 0.71%, and 95.17 ± 0.41%, respectively. ANOVA was conducted to assess the differences in accuracy among the models, revealing statistically significant differences in performance. The results of the ANOVA are presented in Table 3. To further investigate the significant differences identified by ANOVA, Tukey’s Honestly significant difference (HSD) test was performed. The results are presented in Table 4. The analysis revealed that the performance differences between the 3-Layer CNN model and all the other models (DenseNet121, MobileNetV2, and EfficientNetB0) were statistically significant (*p* < 0.05). However, no significant differences were observed in the performance between DenseNet121 and MobileNetV2 (*p* = 0.87), DenseNet121 and EfficientNetB0 (*p* = 0.15), or MobileNetV2 and EfficientNetB0 (*p* = 0.49).

Significant differences are highlighted, indicating that the models showed statistically significant differences in performance.

**Figure 4:**
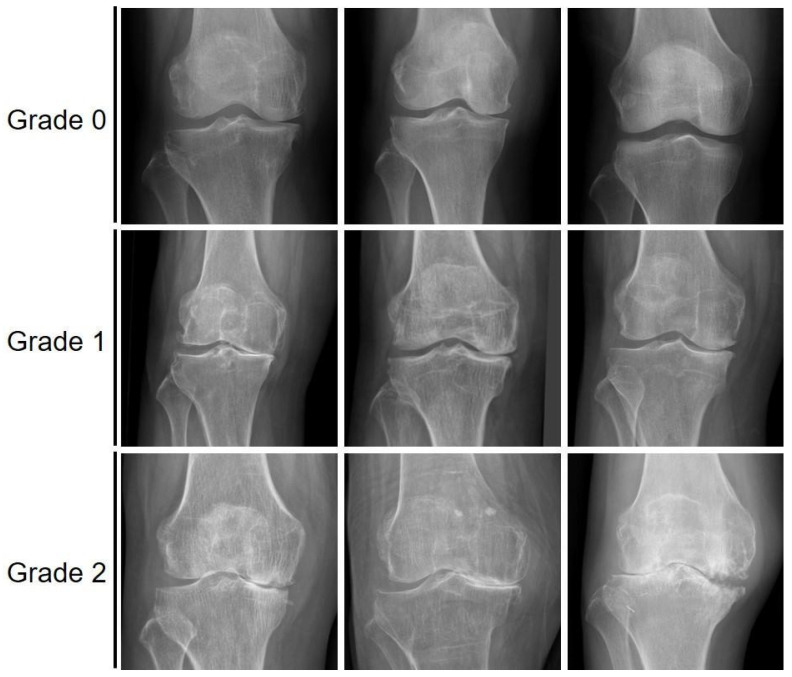
Images where the actual grades match the predicted grades classified by the EfficientNetB0 model.

**Figure 5:**
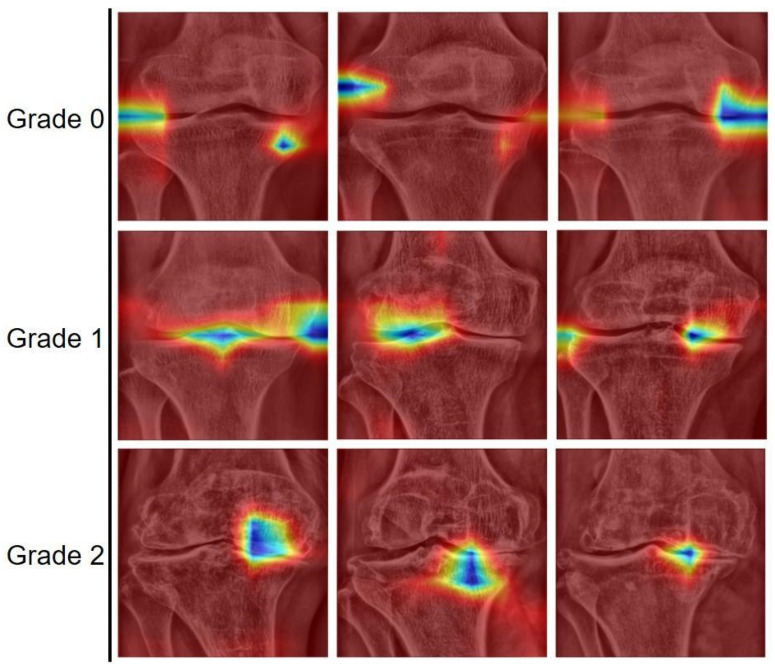
Grad-CAM results for randomly selected test dataset images. This visualization highlights the features of joint structures related to subchondral sclerosis. In our visualization, cooler colors such as blue indicate higher importance, while warmer colors such as red and yellow denote lower relevance, according to the color scale applied.

To analyze the results, the cases predicted by the models were sampled. Various examples of correctly classified subchondral sclerosis grades are shown in Figure 4. Figure 5 shows the image regions that the model focused on for classification using Gradient-weighted class activation mapping (Grad-CAM). Grad-CAM activation was observed in areas prone to subchondral sclerosis, allowing visual confirmation of the joint structural features associated with subchondral sclerosis.

**Figure 6:**
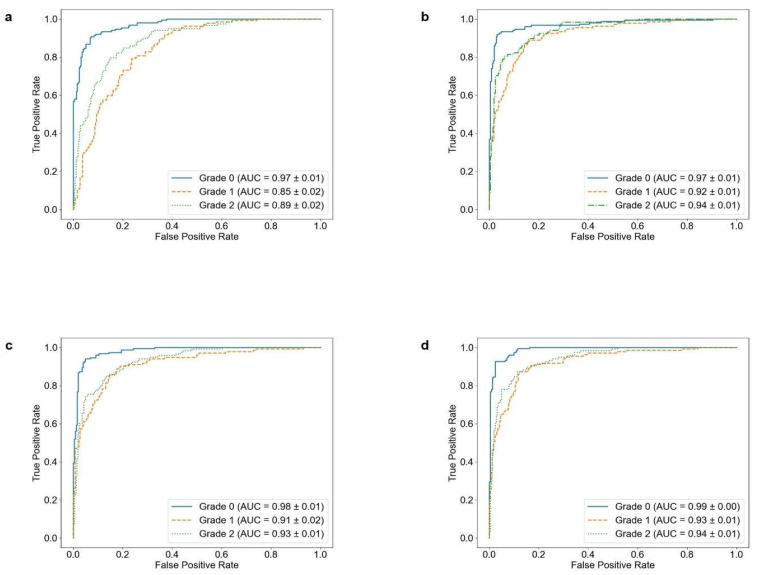
ROC curves for multiclass classification models derived from test data. (**a**) 3-Layer CNN, (**b**) DenseNet121, (**c**) MobileNetV2, and (**d**) EfficientNetB0.

**Figure 7:**
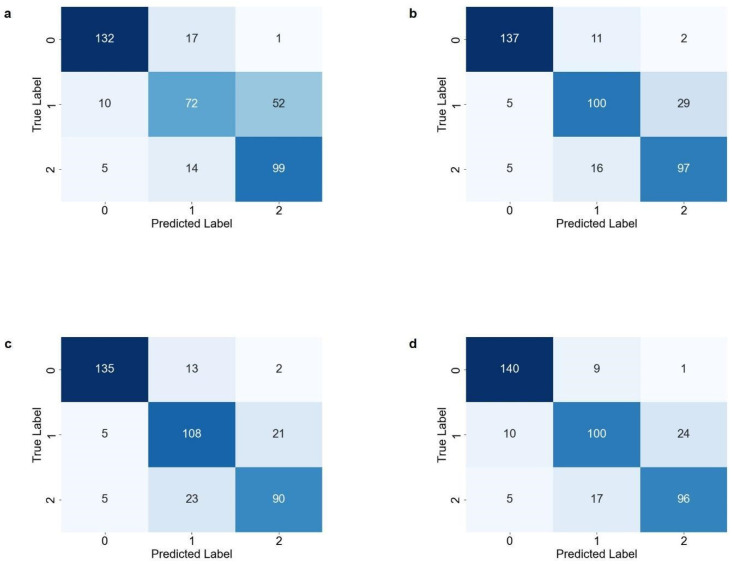
Confusion matrices for multiclass classification models derived from test data. (**a**) 3-Layer CNN, (**b**) DenseNet121, (**c**) MobileNetV2, and (**d**) EfficientNetB0.

Confusion matrices and receiver operating characteristic (ROC) curves were derived using the final test dataset to visually analyze the performance of the models in classifying subchondral sclerosis grades. The results are presented in Figure 6 and Figure 7.

The detection accuracy of the EfficientNetB0 model was compared based on age and sex. The age groups were divided into Group A (individuals in their 20s to 50s) and Group B (individuals in their 60s to 90s), and sex was categorized into Group C (men) and Group D (women). To ensure a fair assessment despite the data imbalance, the macro average accuracy was computed for each group. The results were as follows: the accuracy for age Groups A and B was 0.77 ± 0.09 and 0.84 ± 0.04, respectively, and that for men (Group C) and women (Group D) was 0.78 ± 0.10 and 0.84 ± 0.04, respectively. The results are summarized in Table 5. To determine whether the differences in accuracy between age groups A and B and sex groups C and D were statistically significant, Mann–Whitney U tests were conducted. The 12 results indicated statistically significant differences between the two groups (*p* < 0.05).

## 4. Discussion

This study proposes a novel method for evaluating the subchondral sclerosis grades based on knee radiographs using AI. A majority of studies have focused on knee joint grading or cartilage detection, with research on subchondral sclerosis typically addressing only its presence or absence. In contrast, this study utilized radiographic images collected from hospitals, incorporating diverse data from both men and women aged 6–96 years. It refines the grading of subchondral sclerosis into Grades 0, 1, and 2 for a more precise classification. Various AI models, including 3-Layer CNN, DenseNet121, MobileNetV2, and EfficientNetB0, were developed and compared. The EfficientNetB0 model demonstrated the best performance with an AUC of 95.17 ± 0.41% and an accuracy of 84.70 ± 0.98%. The ANOVA and Tukey’s HSD tests confirmed that the EfficientNetB0 model significantly outperformed the other models. Analysis of model performance by age group and sex using the Mann–Whitney U test revealed statistically significant differences (*p* < 0.05). In particular, the accuracy for women was 0.84 ± 0.04, and that for individuals in their 60s to 90s was also 0.84 ± 0.04. The difference in accuracy according to age was likely due to degenerative changes. In the 60–90 years age group (Group B), a higher incidence of arthritis and more advanced cartilage damage and sclerosis resulted in more distinct subchondral sclerosis lesions on radiographs. Conversely, in the 20–50 years age group (Group A) the relatively less cartilage damage suggests that subchondral sclerosis is less advanced, potentially leading to lower model detection accuracy. This supports the notion that the progression of subchondral sclerosis with age is reflected in the radiographic images [36]. Differences in accuracy by sex may be attributed to physiological changes, such as decreased bone density following menopause, which can accelerate cartilage damage in women and make subchondral sclerosis features more pronounced on radiography. This finding aligns with previous research on the relationship between knee OA and hormonal changes in postmenopausal women [37]. Subchondral sclerosis lesion characteristics vary according to age and sex, and the model can reflect these population characteristics. Von Schacky et al. [17] graded hip OA using deep learning models but only assessed the presence of subchondral sclerosis. In contrast, this study used actual patient data to subdivide subchondral sclerosis into grades 0, 1, and 2 for a more detailed evaluation. Additionally, while Patron et al. [16] used the OAI and MOST datasets, this study employed hospital data to develop a model suited to clinical environments. Mohammed et al. [22] classified OA severity into three grades; however, this study utilized the distinct radiographic features to classify subchondral sclerosis more precisely. Fei, M et al. [26] developed a regression-based model using EfficientNet to predict continuous KL scores, achieving an AUC of 0.83. However, their model revealed inconsistencies between KL labels and radiographic findings, such as visible sclerosis in KL 0 cases. In contrast, our model directly targets subchondral sclerosis as an independent feature, enabling a more precise evaluation of structural changes. As a result, it achieved an AUC of 95.17 ± 0.41%. Tiulpin et al. [15] achieved a multi-class accuracy of 66.71% and an AUC of 0.93 using probabilistic KL grade prediction with joint localization. In contrast, our model focuses on subchondral sclerosis as an independent feature, achieving a higher accuracy of 84.70 ± 0.98% and improved diagnostic specificity.

This study has several limitations. First, the amount of data used is relatively limited. The number of cases used (approximately 4000) may be insufficient for model training and generalization. Future research should collect a larger and more diverse set of patient data to improve model performance and data representativeness. Second, in this study, the CLAHE technique was applied to enhance image contrast, and all experiments were conducted using CLAHE-processed images to ensure consistent experimental conditions. As a result, the model was not evaluated using original images without CLAHE processing. This may be considered a limitation, and future research should include performance comparisons using original images as well. Third, to improve model generalization, it is necessary to collect data that are balanced across sex and age groups and to include radiographic images acquired from a variety of imaging equipment. This will be an important factor in assessing model performance and enhancing its reliability in clinical settings. Finally, the development of an automated system for detecting and classifying subchondral regions in knee radiographic images is required. Addressing these limitations requires further research and additional development.

## 5. Conclusions

This study demonstrates the potential of a radiograph-based classification model for subchondral sclerosis and proposes a novel approach for detailed grading of subchondral sclerosis using artificial intelligence. The EfficientNetB0 model achieved high accuracy (84.70 ± 0.98%) and AUC results (95.17 ± 0.41%) through 10-fold cross-validation, showing superior performance compared to that of existing studies. This indicates that the model has the potential to deliver reliable performance in clinical settings. Future research involving larger datasets and diverse models could further optimize the performance of AI-based classification models for subchondral sclerosis. This will enable a more objective and reliable classification of subchondral sclerosis and advance the development of systems that support clinical decision-making and diagnostic processes.

## Figures and Tables

**Table 1:** Data partitioning of training, testing, and validation subsets for subchondral sclerosis grading.

Subchondral Sclerosis Grade	Train		Validation		Test	
	Sample, *n*	Ratio, %	Sample, *n*	Ratio, %	Sample, *n*	Ratio, %
Grade 0	1178	36.64	142	35.32	150	37.31
Grade 1	1011	31.45	137	34.08	134	33.33
Grade 2	1026	31.91	123	30.60	118	29.36
Total	3215	100	402	100	402	100

Note: This table outlines the sample sizes and ratios for different grades of subchondral sclerosis across the training, validation, and test datasets.

**Table 2:** Distribution of subchondral sclerosis grades in the test data set, categorized by age group and sex.

	Grade 0	Grade 1	Grade 2	Total (Men, Women)	Proportion (%)
20s	4	0	0	4 (0, 4)	1.00
30s	8	0	0	8 (4, 4)	2.01
40s	15	1	0	16 (2, 14)	4.02
50s	42	11	7	60 (17, 43)	15.10
60s	43	42	44	129 (21, 108)	32.39
70s	31	68	54	153 (19, 134)	38.53
80s	6	11	11	28 (3, 25)	7.04
90s	1	1	2	4 (1, 3)	1.00
Total	150	134	118	402	100

Note: This table presents the distribution of different subchondral sclerosis grades in the test dataset, including breakdown by age and sex.

**Table 3:** Performance evaluation results of models.

Model	Sensitivity (%)	Specificity (%)	Accuracy (%)	AUC *	*p*-Value **
3-Layer CNN	73.38 ± 1.38	87.12 ± 0.70	73.81 ± 1.54	89.52 ± 0.46	<0.05
DenseNet121	82.98 ± 1.55	91.75 ± 0.78	83.31 ± 1.62	94.68 ± 0.84	
MobileNetV2	83.41 ± 1.20	91.98 ± 0.61	83.78 ± 1.22	94.45 ± 0.71	
EfficientNetB0	84.27 ± 1.03	92.46 ± 0.49	84.70 ± 0.98	95.17 ± 0.41	

* AUC: area under the curve. ** The *p*-value was calculated using analysis of variance.

**Table 4:** Tukey’s HSD test results for comparison of model performance.

	3-Layer CNN	DenseNet121	MobileNetV2	EfficientNetB0
3-Layer CNN	1.00	<0.001	<0.001	<0.001
DenseNet121	<0.001	1.00	0.8741	0.1523
MobileNetV2	<0.001	0.8741	1.00	0.4892
EfficientNetB0	<0.001	0.1523	0.4892	1.00

*p*-value was calculated using Tukey’s HSD test.

**Table 5:** Comparison of detection accuracy for subchondral bone sclerosis by age group and sex using the EfficientNetB0 model.

	Category	Grade 0		Grade 1		Grade 2		Accuracy (%)	*p*-Value *
		Detected	Total	Detected	Total	Detected	Total		
Age Group	Group A (20s–50s)	63	69	10	12	4	7	0.77 ± 0.09	<0.05
	Group B (60s–90s)	77	81	90	122	92	111	0.84 ± 0.04	
Sex	Group C (Men)	37	38	8	12	12	17	0.78 ± 0.10	<0.05
	Group D (Women)	103	112	92	122	84	101	0.84 ± 0.04	

* *p*-value was calculated using the Mann–Whitney U test. The accuracy values are presented as standard deviations, and the *p*-values indicate statistical significance.

## Data Availability

The data presented in this study are available from the corresponding author upon reasonable request. The data are not publicly available due to privacy and ethical restrictions.
